# An Extrudable Partially Demineralized Allogeneic Bone Paste Exhibits a Similar Bone Healing Capacity as the “Gold Standard” Bone Graft

**DOI:** 10.3389/fbioe.2021.658853

**Published:** 2021-04-20

**Authors:** Pierre Tournier, Jérôme Guicheux, Arnaud Paré, Joëlle Veziers, Ana Barbeito, Raphaël Bardonnet, Pierre Corre, Valérie Geoffroy, Pierre Weiss, Alexis Gaudin

**Affiliations:** ^1^INSERM, UMR 1229, RMeS, Regenerative Medicine and Skeleton, ONIRIS, Université de Nantes, Nantes, France; ^2^BIOBank SAS, Lieusaint, France; ^3^INSERM, UMR 1229, RMeS, Regenerative Medicine and Skeleton, CHU Nantes, ONIRIS, Université de Nantes, Nantes, France; ^4^SC3M Facility, CNRS, INSERM, UMS, Structure Fédérative de Recherche François Bonamy, Université de Nantes, Nantes, France; ^5^Service de Chirurgie Maxillo-Faciale, Plastique et Brulés, Hôpital Trousseau, CHU de Tours, Tours, France

**Keywords:** allogeneic bone, bone graft, bone healing, rat calvaria, pre-clinical

## Abstract

Autologous bone grafts (BGs) remain the reference grafting technique in various clinical contexts of bone grafting procedures despite their numerous peri- and post-operative limitations. The use of allogeneic bone is a viable option for overcoming these limitations, as it is reliable and it has been widely utilized in various forms for decades. However, the lack of versatility of conventional allogeneic BGs (e.g., blocks, powders) limits their potential for use with irregular or hard-to-reach bone defects. In this context, a ready- and easy-to-use partially demineralized allogeneic BG in a paste form has been developed, with the aim of facilitating such bone grafting procedures. The regenerative properties of this bone paste (BP) was assessed and compared to that of a syngeneic BG in a pre-clinical model of intramembranous bone healing in critical size defects in rat calvaria. The microcomputed tridimensional quantifications and the histological observations at 7 weeks after the implantation revealed that the *in vivo* bone regeneration of critical-size defects (CSDs) filled with the BP was similar to syngeneic bone grafts (BGs). Thus, this ready-to-use, injectable, and moldable partially demineralized allogeneic BP, displaying equivalent bone healing capacity than the “gold standard,” may be of particular clinical relevance in the context of oral and maxillofacial bone reconstructions.

## Introduction

Autologous bone grafting is widely used in numerous bone surgical procedures such as the treatment of deformities, traumatisms, non-union fractures, and bone augmentation before dental implant placement (Shegarfi and Reikeras, 2009; Faour et al., 2011). Despite its good success rate [more than 95% graft survival depending on the grafted site (Sakkas et al., 2017)], this technique exhibits numerous peri- and post-operative drawbacks such as bone fracture, infection, pain, blood loss, hematoma, nerve injury, the limited amount available, as well as a longer operating time, which lead to an increase in the final cost (Kurz et al., 1989; Summers and Eisenstein, 1989; Younger and Chapman, 1989; Banwart et al., 1995; Hu et al., 1995; Arrington et al., 1996; Faour et al., 2011; Oryan et al., 2014). Moreover, due to the lack of malleability and versatility, the harvested bone often needs to be reshaped or crushed to fill irregular or hard-to-reach operating sites.

Allogeneic bone is a reliable alternative to autologous bone and it is increasingly being used in numerous indications such as oral surgery (e.g., sinus floor elevation, horizontal/vertical augmentation, alveolar socket preservation) (Kinaci et al., 2014; Malinin et al., 2014; Elakkiya et al., 2017), foot/hand surgeries, arthroplasties or vertebral fusion (Liao et al., 2016; Tuchman et al., 2017). In these clinical indications, allogeneic bone grafts (BGs) exhibit the same bone healing capacities as autologous BG (Del Fabbro and Testori, 2004; Motamedian et al., 2016; Hernigou et al., 2017; Sakkas et al., 2017; Ozgul et al., 2018). Moreover, the use of allogeneic bone displays several advantages over autologous bone. The duration of the surgery is shortened without the need for a bone harvesting procedure, the amount of allogeneic bone available is greater than with autologous bone, as it is collected from operating waste (e.g., femoral heads after total hip replacement surgery), and it can be processed into a variety of sizes and shapes such as blocks, powder, chips, or paste. Structural allografts provide structural and mechanical stability and are mainly used in traumatic reconstructions, osteotomies or arthrodesis, while particulate allografts are more frequently used to fill metaphyseal bone defects or to serve as graft extenders.

In addition to these advantages, allogeneic bone tissues can be used as such or demineralized in order to improve their regenerative potential (Urist, 1965; Wood and Mealey, 2012). The main advantages of demineralized BGs lie in their abundance, the possibility to obtain a moldable or extrudable material that has substantial bone healing capacities, leading to dozens of clinically available products (Delloye et al., 2007; Gruskin et al., 2012). Yet, such grafts can exhibit variable bone healing properties, considering the donor variation and often requires to be mixed with an additional product which can be deleterious for bone healing (Pacaccio and Stern, 2005; Baldwin et al., 2019; Cho et al., 2020). Moreover, few studies compared their bone healing properties to gold standard autografts, and they have poor mechanical properties without the possibility of radiological monitoring.

In light of this, improvement of the demineralization (HCl baths), sterilization (gamma rays, autoclaving), and cleaning processes (NaOH baths, supercritical CO_2,_ sonication) of the bone tissues (Froum et al., 2002; Wood and Mealey, 2012; Eagle et al., 2015; Chadwick et al., 2016; Whetman and Mealey, 2016; Narotzky et al., 2020) are important to limit the variable outcomes in terms of bone healing that are largely due to the very different procedures and associated with alteration of the bone tissue (Rasch et al., 2019). In order to combine the radio-opacity of mineralized BGs and the regenerative properties of demineralized BGs, partially demineralized BGs have been developed in the past decades in block forms (Hallfeldt et al., 1995). However, this type of partially demineralized graft exhibits limited bone healing properties and handling capacity, leaving the challenges of this combination unmet.

To address these challenges, a novel partially demineralized allogeneic BG that allows radiological follow-up, easy handling, rapid use, and versatility in terms of shape adaptability, in addition to exhibiting a substantial bone healing capacity, is described in this work. This allogeneic BG is in a paste form, consisting of partially demineralized bone powder particles, engulfed in collagen-rich gelatin, specifically indicated for irregular or hard-to-reach non-load-bearing areas (Bardonnet and Barbeito, 2015). In light of its handling characteristics, we believe that this type of BP could be a promising alternative to the use of autologous BGs if it can be shown to exhibit similar bone-healing properties. Therefore, a preclinical evaluation of the regenerative properties of this BP comprising a comparison with the “gold standard” BG is required before a clinical transfer can be considered.

Thus, the purpose of this work was to assess the *in vivo* regenerative properties of this allogeneic BP in a preclinical model of intramembranous bone healing [i.e., calvarial critical-size defects (CSDs) in a syngeneic rat strain] and compare to that of a syngeneic BG.

## Materials and Methods

### Animals and Ethical Aspects

All of the procedures involving animals were conducted in accordance with the institutional guidelines of the European and French Ethics Committee and they were approved by the local ethics committee (Comité d’éthique en Expérimentation animal, Pays-de-la-Loire (CEEA.PdL.06); authorization n° 2019012313017323 / APAFIS 18616). Concerted and special efforts were made to minimize psychological and physical suffering as well as to reduce the number of animals used. Thirty-three seven-week-old male syngeneic Lewis 1A RT1a-haplotype rats were purchased from an approved breeder (Charles River, Écully, France). Twenty-four animals were randomly assigned to the following experimental groups: empty defect (control), bone graft (BG), or bone paste (BP), with three time points (0, 3, and 7 weeks). Two animals were included in each group at 0 weeks, and three animals at 3 and at 7 weeks. Two defects were generated per animal, for a total of four defects at 0 weeks and six defects at 3 and at 7 weeks per experimental group. Nine animals were used as syngeneic bone graft donors.

### Calvarial Critical-Size Defect Surgical Procedure

**FIGURE 1:**
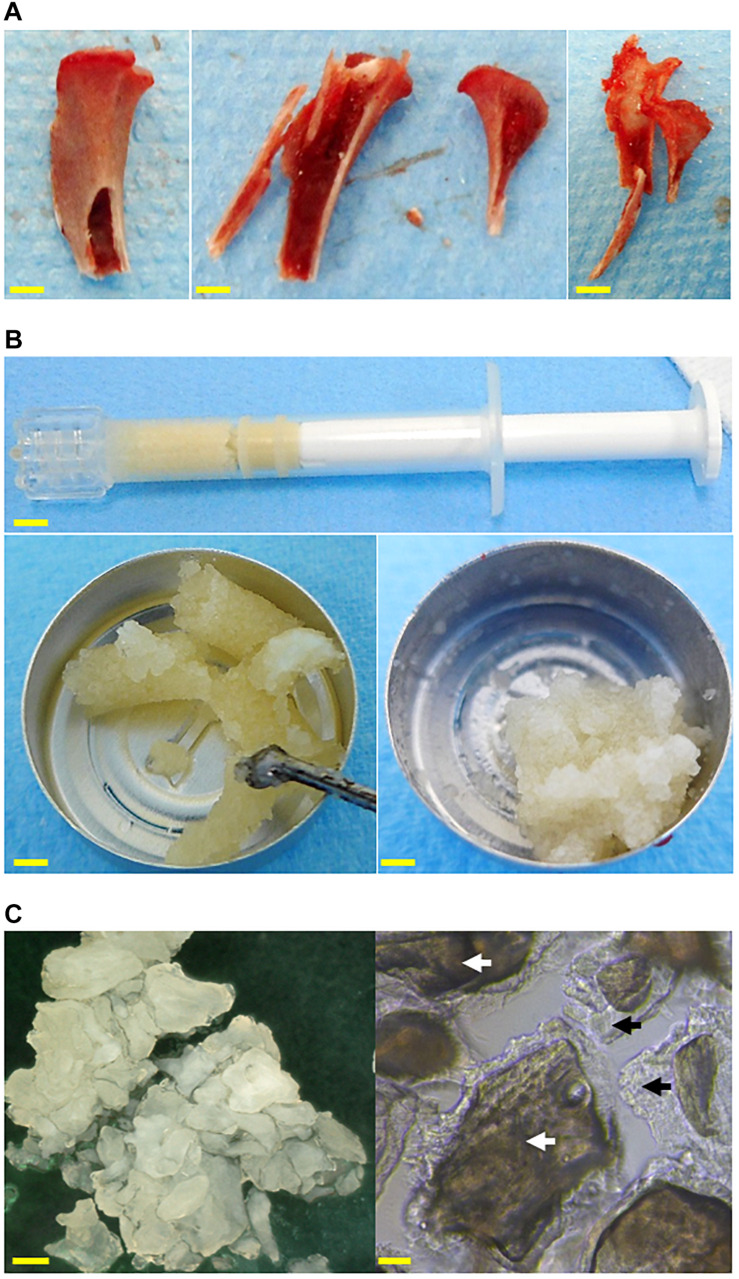
Images of the grafts used to fill the calvarial critical bone defects. **(A)** Syngeneic bone graft harvested in a proximal tibia, from left to right: before being cut, after being cut, and after bone harvesting. **(B)** The bone paste in a syringe (top, scale bar: 5 mm) after extrusion (left, scale bar: 3 mm), and after modeling (right, scale bar: 3 mm). **(C)** Microscopic observations of the bone paste particles in air (left, reflected light, scale bar: 300 μm) or in water (right, transmitted light, scale bar: 50 μm). Mineralized core: white arrows, demineralized bone matrix: black arrows.

All of the veterinary medicines were obtained from Centravet (Dinan, France). The animals were allowed to acclimate for a week at the animal facility before the surgery. The acclimated animals were anesthetized by inhalation of an isoflurane/air mixture (4% isoflurane) in a closed induction chamber and then placed on a heating pad with an isoflurane/air mixture (2% isoflurane) through an inhalation mask throughout the surgical procedures. The creation of the critical-size defects (CSDs) was carried out as previously described (Spicer et al., 2012). Briefly, the top of the skull was shaved and the skin was cleaned with sterile water and iodized polyvidone (Betadine). Peri- and post-operative analgesia was ensured by subcutaneous infiltration of lidocaine (Xylocaine, 5 mg/kg) on the operating site, buprenorphine (Buprecare, 0.02 mg/kg twice daily for 3 days), and meloxicam (Metacam, 1 mg/kg) in the rear of the animals. The skin and periosteum were incised and pushed back on the sides with the skin and has been put back after the surgery. Bilateral full thickness parietal bone defect on the top of the skull were then performed using a 5 mm outer diameter trephine, resulting in 5 mm gaps. The operating sites were rinsed abundantly with saline throughout the surgery to avoid cauterization of the edges of the defects. The defects were left unfilled for the controls or they were filled with BG or BP. The BG were obtained from additional animals euthanized in a CO_2_ closed chamber as previously described (Corre et al., 2015). Briefly, the humeri, femurs, and tibiae were harvested, cut lengthwise, and the trabecular bone was collected by scraping (Figure 1A) and then immediately implanted. The bone harvested from a single donor was sufficient to fill two defects. The skin was then sutured [5/0, non-absorbable suture (Ethicon, Bridgewater, United States)] and the animals were returned to their cages with water and food *ad libitum*. At the appropriate time points, the animals were euthanized in a CO_2_ chamber. The calvaria were collected using sharp scissors inserted through the *foramen magnum*, and the skulls were cut following the temporal crest to the frontal bone, above the coronal suture. The calvaria were fixed in 4% paraformaldehyde for 72 h and then stored in 70% ethanol.

### The Process to Obtain the Bone Paste

The allogeneic bone paste (BP) was obtained from human donors who had provided their informed consent, and all of the protocols were approved by the French ethical committee “Comité de Protection des Personnes” (CPP), in accordance with the 1975 Helsinki declaration and its subsequent amendments. The bone tissues were harvested by a specialized bone tissue bank (BIOBank, Lieusaint, France) under the authorization of the “Agence Nationale de Sécurité du Médicament et des produits de santé” (ANSM, n°FR07703T-19-01) according to the French regulations. The femoral heads were cleaned, as previously described, by supercritical CO_2_ lipid extraction and successive immersions in H_2_O_2_, NaOH, and EtOH (Fages et al., 1994, 1998; Bardonnet, 2005). The cleaned femoral heads were crushed into particles of bone powder (0.3–1 mm in diameter). The BP is produced by the partial demineralization of the bone powder with HCl 0.4 N for 1 h–1 h 30 under stirring. These partially demineralized particles were hydrated with H_2_O and the outer demineralized bone matrix of the particles was heat-denatured by autoclaving (134°C, 18 min) as previously described (Bardonnet and Barbeito, 2015), allowing sterilization of the grafts. This preparation process allowed a cohesive, extrudable, and moldable BP to be obtained that was composed of particles consisting of a mineralized core surrounded by demineralized bone matrix engulfed in collagen-rich gelatin (Figures 1B,C). All of the samples used in his study were made from donor pools so as to minimize the inter-donor variability.

### Radiological Acquisitions and Quantifications

The samples were scanned with a SkyScan-1072 device (Bruker, Billerica, United States) under the following conditions: 70 kV, 142 μA, resolution = 18 μm, and single 360° scan. Three-dimensional datasets were reconstructed with NRecon software (Micro Photonics Inc., Allentown, United States). The bone volume (BV) and the tissue volume (TV) within the defects were quantified with the CTAn software. Tree-dimensional quantitative results were expressed as BV/TV (%) at 0, 3, and 7 weeks.

### Histological Analyses

The histological samples were processed by Novotec (Bron, France). Briefly, the samples were demineralized with an HCl solution for 7 h (R.D.O., Eurobio, France), then dehydrated in graduated baths of ethanol, acetone, and xylene, followed by embedding in paraffin. Frontal sections with a thickness of 5 μm were generated with a microtome and then stained with Harris hematoxylin, eosin, and saffron (HES) and mounted in Entellan^®^ (Merck, Burlington, MA, United States). The stained slides were observed with an optical microscope (Leica DM2000) and images were taken with a digital camera (Leica DFC420C) using image acquisition software (LAS V4.2).

### Statistical Analyses

The statistical analyses were performed using GraphPad Prism 5.0 software (GraphPad, San Diego, United States). Statistical significance was determined using one-way ANOVA followed by Bonferroni’s *post hoc* test for multiple group comparisons. Statistical significance was set at *p* < 0.05. The results are presented as means ± SD. The normality of the data distribution has been verified thanks to a Shapiro-Wilk test with a *P* < 0.05.

## Results

The regenerative potentials of the BG and the BP were first investigated with micro-computed tridimensional analyses after implantation in a rat calvarial critical-size defect model.

**FIGURE 2:**
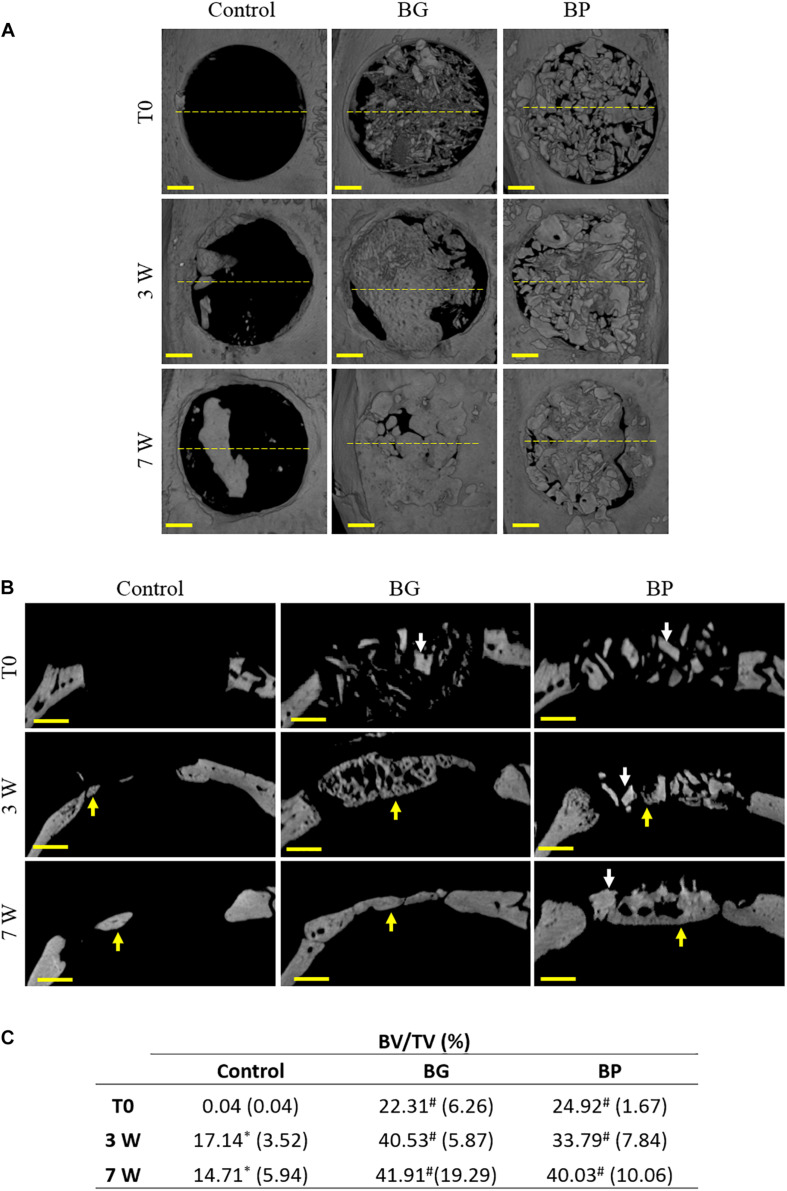
Radiological observations and tridimensional quantifications of bone healing in critical-size defects in rat calvaria. Representative images of the 3D reconstruction of the defects at various times after the surgery (immediately after surgery (T0), 3 weeks (3 W), and 7 weeks (7 W) after surgery). The defects were left unfilled (control), filled with a syngeneic bone graft (BG), or filled with the bone paste (BP). **(A)** Top views and **(B)** coronal sections are shown. The yellow dashed lines indicate the coronal sections. Arrows indicate the grafted particles (white) or the newly formed bone (yellow). Scale bars: 1 mm. **(C)** Tridimensional quantifications of the bone volume (BV) relative to the tissue volume (TV) in the defects. ^∗^*p* < 0.05 vs. T0 (columns), ^#^*p* < 0.05 vs. Control (rows) (ANOVA with Bonferroni’s *post hoc* test).

For the grafted defects, the radiological images allowed the size and shape of the BG and BP particles to be imaged after their implantation (T0). The BG consisted of a blend of highly variably sized particles produced by grinding the trabecular bone by hand. On the other hand, the BP particles were more uniform in size (Figures 2A,B white arrows).

As expected in this model, 3 weeks after implantation, the control defects exhibited very limited new bone formation, which was mainly localized at the edges of the defects (Figures 2A,B, yellow arrows). In the BG-grafted defects, the particles were no longer distinguishable from the newly formed bone, which could be seen at the edges and in the center of the defect (Figures 2A,B yellow arrows). However, the newly formed bone was found to be inhomogeneous and it exhibited numerous lacunae. Conversely, at this time point the BP particles were still discernible (Figures 2A,B, white arrows), with newly formed bone at the edges and in the center of the defects (Figures 2A,B yellow arrows), albeit to a lesser extent compared to the BG-grafted defects.

Seven weeks post-surgery, the amount of bone tissue in the control defects was still very limited and located at the edges of the defect (Figures 2A,B yellow arrows). The bone regrowth in the BG-grafted group was evidenced by compact bone tissue that was distributed homogeneously in the defects (Figures 2A,B yellow arrows). In the BP-grafted group, the particles were harder to discern compared to the previous time point (Figures 2A,B white arrows) due to their integration into the regenerated bone and because their radio-opacity was similar to that of the newly formed bone tissue. Nonetheless, at this time point, a significant volume of newly formed bone was observable in these defects (Figures 2A,B yellow arrows).

In addition to those observations, the tridimensional quantification of the BV/TV in the defects yielded equivalent values in the BG- and the BP-grafted groups at the time of the surgery, thus indicating that the same volume of graft had been implanted in the defects. After 3 and 7 weeks of regeneration, the BV/TV values were again equivalent in the BG- and the BP-grafted defects, and they were also always significantly higher than in the control group (Figure 2C). The mean values of BV/TV in the BG- and the BP-grafted defects roughly doubled from the time of implantation to 7 weeks post-implantation. However, due to the substantial variability in the values in both groups, the differences between these time-points were not statistically significant (Figure 2C).

The overall appearance and the quality of the regenerated bone tissue were then assessed based on histological analyses of the defects at all of the time points, and these observations confirmed the quantitative radiological analyses.

**FIGURE 3:**
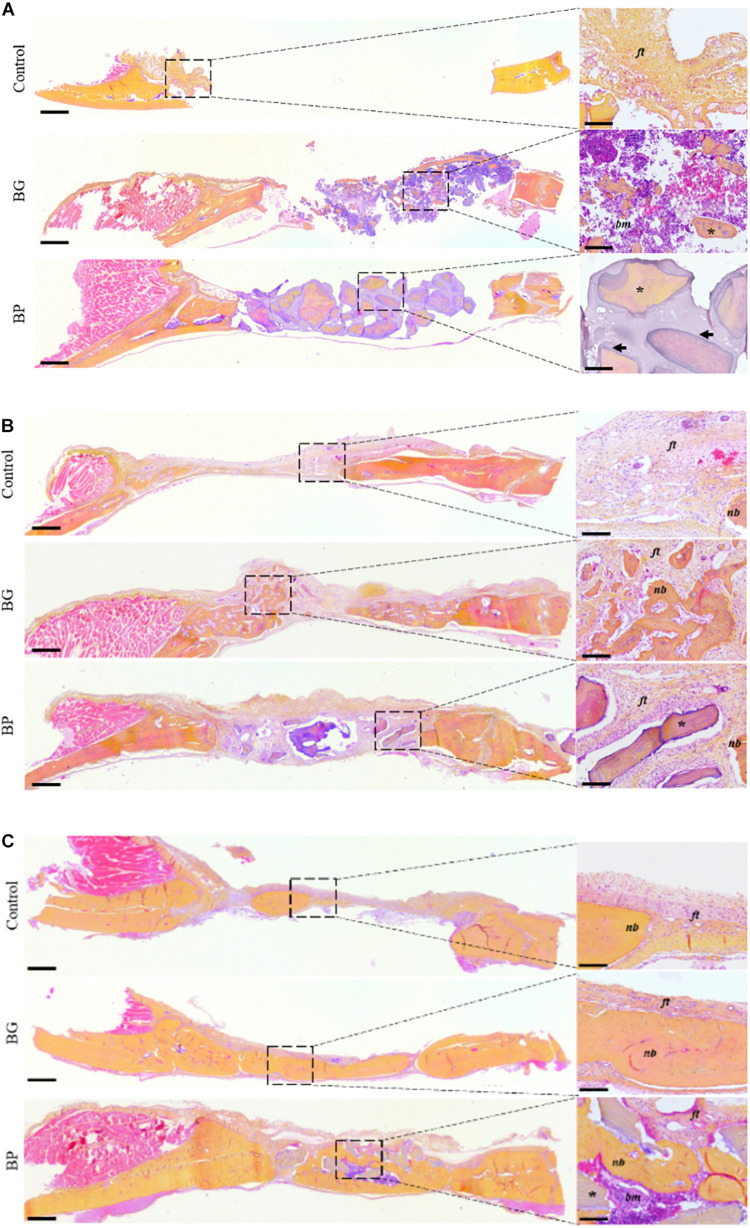
Histological observation of the bone regeneration in a critical-size defect model in rat calvaria. Representative images of the defects by HES staining of decalcified 5 μm-thick frontal sections of the defects after surgery **(A)** T0, **(B)** 3 weeks, and **(C)** 7 weeks. The defects were left unfilled (controls), filled with a syngeneic bone graft (BG), or filled with the bone paste (BP). Scale bars: 500 μm for the full defects, 100 μm for the enlarged sections (dashed square). Dark arrows: demineralized bone matrix, dark yellow: collagen-rich bone tissue, light red: cell cytoplasm, purple/blue: nuclei, *ft*, fibrous tissue; *nb*, new bone; *, bone grafts; *bm*, bone marrow.

Immediately after the implantation, the irregular size of the BG-grafted particles was observable (Figure 3A, black star) along with the bone marrow (Figure 3A, *bm*). Once implanted, the BP particles could be discerned based on their inner mineralized core (Figure 3A, black star) and outer demineralized bone matrix (Figure 3A, black arrow). Moreover, the gelatin-rich denatured bone matrix produced by the heat denaturation could be seen between the particles.

After 3 weeks of implantation, a very limited amount of newly formed bone tissue was observable in the control defects, and these were predominantly filled with a fibrous tissue (Figure 3B, *ft*). In the BG-grafted defects, the implanted particles had become integrated into the newly formed bone and they were, therefore, hard to discern. The implanted bone marrow had become replaced by a fibrous tissue (Figure 3B, *ft*), and a lacunar woven bone tissue had grown from the edges of the defects toward their center. At this time point, the BP particles could still be discerned in the defects, but the denatured gelatin-rich bone matrix between the particles was no longer visible (Figure 3B). In this group, the particles were encapsulated by a fibrous tissue, and the newly formed bone was mainly located at the edges of the defects.

Seven weeks after implantation, a very limited degree of bone formation was observed in the control defects (Figure 3C, *nb*), with an abundant fibrous tissue (Figure 3C, *ft*). In the BG-grafted defects, the particles were no longer distinguishable, and a larger amount of newly formed bone could be seen compared to the previous time point (Figure 3C, *nb*). This bone tissue consisted of an association of woven and lamellar bone, thus suggesting that tissue remodeling had taken place in the grafted defects. This remodeled tissue contained numerous osteocytes and a number of blood vessels, similar to the native bone tissue outside the defects. Analogously, a small amount of fibrous tissue remained in the BP-grafted group at this time point. The BP particles were still observable in the defects, with the empty osteoplasts in the mineralized inner core of the particles.

Moreover, the newly formed bone tissue displayed a comparable organization and composition as that of the BG-grafted group, i.e., an association of woven and lamellar bone with a collagen-rich matrix, as well as numerous osteocytes and a number of blood vessels. Additionally, some areas of bone marrow were observable in the BP grafted defects (Figure 3C, *bm*).

## Discussion

The limitations of gold standard bone grafting procedures (i.e., autologous bone grafting) have led to the development of easy-to-use alternative bone grafts (such as allogeneic bone in paste or putty forms) for use in various bone surgical procedures. This work aimed to evaluate the bone regenerative properties of a novel, extrudable, ready- and easy-to-use allogeneic bone graft in a paste form compared to a syngeneic bone graft. Based on our study, the main advantages of this bone paste (BP) lies in its rapid and easy use, combined with its radiological follow-up and substantial bone healing properties.

The use of a syngeneic rat strain (i.e., whereby the donor and the receiver have the same immunological identity) allows bone grafts to be performed without them being subject to rejection. This surgical procedure is, therefore, a model comparable to the clinical gold standard of autologous bone grafting, although it is easier to perform in small animals, such as rats, and it does not have the drawbacks of autologous bone harvesting. Although the rodent model allow a rapid and cost-effective examination of bone healing properties of bone graft materials, its main limitations lies in the limited size of the bone defects, the absence of mechanical stress which can play a role in bone healing, and the remodeling which have different kinetics with the healing of a human bone.

Based on the radiological observations and quantifications, the BG and the BP exhibited comparable bone healing capacities, 7 weeks after their implantation in a rat calvaria CSD. Nonetheless, the bone healing progressed with slightly different kinetics, as shown by the data at 3 weeks after the BG and the BP implantations. The barely observable BG particles in the grafted defects at 3 and at 7 weeks after their implantation either suggested their complete integration into the newly formed bone or their cell-mediated degradation, which is a ubiquitous feature of bone regeneration and turnover (Raggatt and Partridge, 2010; Kylmaoja et al., 2016; Soysa and Alles, 2016).

On the other hand, the BP particles remained discernible at all of the time points, thereby allowing their radiological follow-up in light of their characteristic shape, which is an advantage compared to radio-transparent bone substitutes such as fully demineralized bone matrices. Of note, the denatured gelatin-rich bone matrix between the particles was no longer visible 3 weeks after the BP implantation, presumably due to exposure to the biological fluids. Moreover, the observation of BP particles as part of the newly formed tissue at all of the time points suggests limited resorption of the particles. This outcome is of considerable value as it indicates that bone regrowth may be achieved with only limited degradation of the BP particles, thereby allowing an elevated bone volume (BV) to be rapidly attained during tissue healing. However, due to their similar radiopacities, the particles and new bone are indistinguishable by conventional radiographic approaches. Thus, the radiological quantifications of the BV/TV were calculated by inclusion of both the particles and the new bone. The elevated BV/TV and the gain in bone tissue height in the BP-grafted defects may be a key point in clinical indications such as sinus floor elevation where restoration of the bone height is being sought.

The histological observations confirmed the radiological results, whereby the BG particles were not found in the defects, while the BP were observable both at 3 and at 7 weeks post-implantation. Although the histological observations documented the regeneration of the BG- and the BP-grafted defects, 7 weeks after the implantation, these analyses highlighted the slightly different bone healing kinetics. Indeed, while both the BG- and the BP-grafted groups underwent a phase of cellular infiltration with the presence of a cellular-rich tissue in contact with the particles, a larger amount of new bone tissue formation was observable in the BG-grafted groups at 3 weeks post-surgery, thus suggesting faster regeneration in these defects. This outcome could be explained by the cells and growth factors in the bone marrow associated with the BG particles in the defects. It is nonetheless worth noting in this CSD model that complete bone healing was neither achieved with the BG -as fibrous tissue was still present in the defects- nor with the BP, in which a certain amount of bone marrow was observable. In addition, in a recent study assessing the BP in the same CSD model in rat calvaria, at 3 weeks post implantation, lamellar bone structures with numerous osteocytes and a number of blood vessels could be observed. In this previous work, at 7 weeks post implantation the defects filled with the BP exhibited a limited amount of soft tissue. At this time point, the regenerated bone exhibited a typical lamellar osseous structure, with numerous osteocytes and vasculature reminiscent of that observed in native bone tissue outside the grafted area (Tournier et al., 2021).

The substantial bone healing in these critical-size defects implanted with BP is of particular interest as bone regeneration in this model is a challenge (Corre et al., 2015; Hivernaud et al., 2019; do Lago et al., 2020; Paré et al., 2020) and widely clinically used bone substitutes need to be mixed with bone marrow aspirate or growth factors to heal such defects. The bone regeneration observed with the BP is a promising result that is reason for further clinical investigation of its regenerative properties in critical and subcritical defects compared to the gold standard of autologous bone grafting.

Longer time points are needed to assess whether full bone healing can be obtained, and this could also provide an indication of the longevity of the BP-grafted particles in the regenerated bone, especially if osteoclast-mediated degradation of these particles takes place as part of the bone turnover.

Nonetheless, the investigation of the bone regeneration mechanisms triggered by the BP remains to be fully elucidated. The inductive effect of our partially demineralized BP has not been established yet, and the contribution of the degree of demineralization to bone healing is also to be answered. The demineralization of bone matrix has been shown to result in the release of growth factors that induce bone regeneration (Urist, 1965; Urist et al., 1983). However, the BP preparation process includes a heat-treatment step that is thought to result in denaturation of these growth factors (Ohta et al., 2005), thereby limiting their contribution to bone regrowth. On the other hand, collagen-based bone substitutes have been reported to modulate the phenotype of macrophages, which are key effectors in bone regeneration (Lyons et al., 2010; Elgharably et al., 2014; Sun et al., 2016; Shi et al., 2018), and the outer demineralized bone matrix of the BP particles may interact with monocytes and macrophages to promote their pro-regenerative phenotype. Finally, heat-denatured collagen is known to expose RGD (Arg-Gly-Asp) integrin-binding sites, which promote adhesion and drive the osteogenic fate of bone marrow mesenchymal stromal cells, thereby enhancing bone regeneration *in vivo* (Pfaff et al., 1993; Porté-Durrieu et al., 2004; Sawyer et al., 2005; Chua et al., 2008; Detsch et al., 2010; Taubenberger et al., 2010; Suwa et al., 2016). Therefore, we hypothesize that the bone regenerative capacity of the BP may at least partly be due to the interaction of macrophages and osteoprogenitor cells with the outer partially demineralized surface of the BP particles.

This work strongly suggests that the substantial amount of newly formed bone in the BP grafted defects at 7 weeks after the implantation is comparable to the gold standard and that it can be achieved without the drawbacks of bone harvesting, which is a major advantage for clinical use. Since this BP does not require any mixing, reconstitution, hydration, or incubation prior to its use, it is of substantial interest to clinicians. It allows bone surgery to be undertaken without having to be concerned about bone graft harvesting, preparation, or conservation, in addition to allowing any unexpected issues to be managed more readily. The easy preparation of the BP also allows it to be used without a need for any specific equipment, which is particularly relevant for small medical facilities or in developing countries. Taken together, our data encourage the evaluation of the bone healing properties of the BP in other sites to broaden its applications (e.g., spine, trauma surgery), and its clinical evaluation in oral and maxillofacial bone regenerative medicine.

## Data Availability Statement

The raw data supporting the conclusions of this article will be made available by the authors, without undue reservation.

## Ethics Statement

The animal study was reviewed and approved by the Comité d’éthique en Expérimentation Animal, Pays-de-la-Loire (CEEA.PdL.06); authorization n° 2019012313017323.

## Author Contributions

PT conceptualized the study, performed the experimentations, analyzed the data, wrote, and edited the manuscript. AP and JV performed the experimentations. AB, RB, and PC conceptualized the study. JG, VG, PW, and AG conceptualized the study, wrote, and edited the manuscript. All authors contributed to the article and approved the submitted version.

## Conflict of Interest

PT, AB, and RB were employees of BIOBank. The company had no influence in the study design; the collection, analysis and interpretation of the data; the writing of the manuscript; or the decision to submit the manuscript for publication. The remaining authors declare that the research was conducted in the absence of any commercial or financial relationships that could be construed as a potential conflict of interest.
